# Neighbourhood characteristics, lifestyle factors, and child development: Secondary analysis of the All our families cohort study

**DOI:** 10.3389/fepid.2022.1073666

**Published:** 2023-01-17

**Authors:** A. L. MacKinnon, H. Sell, K. Silang, E. B. Xie, J. W. Jung, S. Tough, L. Tomfohr-Madsen

**Affiliations:** ^1^ Department of Psychiatry and Addictology, Université de Montréal, Montréal, QC, Canada; ^2^ CHU Sainte-Justine Research Center, Montréal, QC, Canada; ^3^ Immunization Programs and Vaccine Preventable Diseases Service, BC Centre for Disease Control, Vancouver, BC, Canada; ^4^ Department of Psychology, University of Calgary, Calgary, AB, Canada; ^5^ Department of Community Health Sciences, University of Calgary, Calgary, AB, Canada; ^6^ Alberta Children's Hospital Research Institute, Calgary, AB, Canada; ^7^ Department of Educational and Counselling Psychology and Special Education, University of British Columbia, Vancouver, BC, Canada

**Keywords:** neighbourhood, lifestyle, child development, parents, community

## Abstract

**Background:**

Neighbourhood characteristics have been found to influence child development, but little is known about lifestyle factors that may moderate this relationship, which can provide modifiable targets for policies and programing. This study investigated the association between neighbourhood characteristics (e.g., deprivation, disorder) during pregnancy and child development at age 5 in relation to various lifestyle factors (e.g., physical activity, parent-child reading, community resource use) during early childhood.

**Methods:**

A secondary analysis was conducted using multilevel modeling of data from the All Our Families cohort, recruited in Canada from 2008 to 2010. Participants self-reported on demographics during pregnancy, lifestyle factors at 3 years, and child development at 5 years using the Ages and Stages Questionnaire (ASQ-3). Neighbourhood deprivation was evaluated using the Vancouver Area Deprivation Index (VANDIX), while disorder was measured using police services' community crime reports.

**Results:**

Geocoded information was available for 2,444 participants. After adjusting for covariates, multilevel modeling indicated a significant negative association between neighbourhood deprivation and overall child development (b = −.726, 95% CI: −1.344, −.120). Parent-child reading was found to be a significant moderator of the effect of neighbourhood disorder (b = .005, 95% CI: .001, .009). There were no statistically significant moderation effects for physical activity or community resource use.

**Conclusion:**

Neighbourhood deprivation during pregnancy is associated with early child development. Parent-child reading may function as a protective factor in the presence of higher neighbourhood disorder. Overall, neighbourhood-level effects should be considered in policies and community programs that promote family and child well-being.

## 1. Introduction

Despite the importance of early childhood development for well-being (1, 2) and the provision of federally funded early childhood education and care (ECEC), many Canadian children remain vulnerable to developmental delays. In Canada, approximately one in four children (26%) are reported to be vulnerable to delays in one or more developmental areas at time of entry into grade one (3). Notably, children in low-income neighbourhoods displayed a higher rate of developmental vulnerability (34.9%) compared to children in high-income neighbourhoods (19.5%) (3). Similarly, living in higher poverty index neighbourhoods in Canada has been associated with significant declines in young children's physical health and well-being (4, 5). Less is known about the impact of neighbourhoods during pregnancy, a critical period for child development, and potential protective factors in the first few years postpartum.

A variety of neighbourhood characteristics have been linked with well-being, and these generally include both physical and social characteristics. Physical characteristics refer to neighbourhood attributes such as degree of urbanization (e.g., density) (6, 7), public and open spaces (e.g., walkability, transportation, cleanliness) (8), available resources and facilities (9), green space (10), environmental noise (e.g., traffic) (11), and air pollution (12, 13). Social characteristics refer to factors such as neighbourhood deprivation (i.e., low socioeconomic status (SES) (14), disorder (i.e., incivility, deterioration, crime) (15), social capital (16), and ethnic composition (12, 17). Extant research has found strong associations between neighbourhood characteristics, particularly deprivation, with physical, behavioural, and mental health outcomes in children (18–21). Children in disadvantaged neighbourhoods (i.e., lower SES and poor physical conditions) on average were at higher odds of experiencing obesity (22), having poor peer relations (23), lower cognitive development (24, 25) and more mental health concerns (26). By contrast, children living in neighbourhoods that are perceived as having higher collective efficacy (i.e., belief in the capability of the community to maintain social order) were more likely to play outside, watch less television, and engage in more activities that promote socialization and physical stimulation (27).

The impact of neighbourhood characteristics during pregnancy is particularly pertinent to explore in light of the Developmental Origins of Health and Disease (DOHaD) hypothesis, which postulates that exposure to certain environmental influences *in utero* may have both short and long-term consequences (28). According to this theory, if a pregnant person is exposed to poor environmental conditions (i.e., neighbourhood deprivation), the fetus may develop adaptations to help immediate survival and future response if a similar environment is encountered again (i.e., down-regulation of metabolic and organ function). However, these adaptations can lead to long-term changes in child development (29). Previous findings from the All our Families cohort study have linked prenatal exposure to neighbourhood deprivation and disorder with child outcomes, including lower language scores at 5 years of age, even after controlling for family history of language delay, infant sex, and early vocabulary (20). Furthermore, greater neighbourhood deprivation has been indirectly associated with poor infant sleep consolidation through perceptions of poor neighbourhood safety and maternal anxiety (30).

It is also important to consider resilience and plasticity of child development despite exposure to prenatal adversity. Social interactive processes and lifestyle at the individual and family level have been proposed as protective factors for the influence of neighbourhood characteristics on child development (31), however, few studies have examined these as moderators. For example, involvement in community programs or activities has been found to mitigate the impact of neighbourhood problems, including violence, on academic performance and depression (32, 33). Potential protective lifestyle factors warranting investigation include physical activity, parent-child reading, and community resource use as they have been previously associated with positive developmental outcomes such as motor skills, vocabulary, and behaviour (34–36). Identifying such lifestyle factors as moderators could provide modifiable targets that help buffer against the impact of adversity during pregnancy and minimize the possibility of long-lasting adverse effects of neighbourhoods.

The current study aimed to investigate: (1) to what extent neighbourhood characteristics (e.g., deprivation and disorder) during pregnancy are associated with developmental outcomes in children at age 5; and (2) how various lifestyle factors (e.g., physical activity, parent-child reading, and community resource use) may moderate the association between neighbourhood characteristics and child development. In terms of hypotheses, children whose birthing parents were living in neighbourhoods with more deprivation and disorder during pregnancy were expected to have poorer development at age 5, whereas positive lifestyle factors (i.e., physical activity, community resource use, parent-child reading) during early childhood were hypothesized to be associated with better development and buffer the impact of neighbourhood characteristics.

## 2. Materials and methods

### 2.1. Study design & procedure

The current investigation utilized data from the larger, ongoing All Our Families (AoF) cohort study (37, 38) in Alberta, Canada. From 2008 to 2010, a total of 4,011 individuals responded to community advertisements or researchers at primary health care offices and laboratory services recruiting “pregnant women” (referred to as birthing parents or participants throughout since gender identity information was not collected), of which 3,387 met eligibility criteria (understand English, >18 years old, <25 weeks' gestation, receiving prenatal care near Calgary, Canada) and were enrolled in the study. All participants provided informed written consent and ethical approval was obtained from the Conjoint Health Research Ethics Board (CHREB) at the University of Calgary for both the original study (REB13-0868) and secondary analysis (REB19-1417). Participants were asked to complete questionnaires twice during pregnancy (<25 and 34–36 weeks' gestation) and were followed up at 4 months and 1, 2, 3, and 5 years postpartum.

### 2.2. Measures

#### 2.2.1. Sociodemographic variables

Participants reported on relevant sociodemographic variables during pregnancy (at <25 weeks of gestation) including their ethnicity, age (years), education (1 = *some elementary to high school*, 2 = *graduated high school*, 3 = *some college/trade/university,* 4 = *graduated college/trade/university,* 5 = *some graduate school*, and 6 = *completed graduate school*), postal code, annual household income (1 = *less than $10,000*, 2 = *$10,000 to $19,999*, 3 = *$20,000 to $29,999*, 4 = *$30,000 to $39,999*, 5 = *$40,000 to $49,999*, 6 = *$50,000 to $59,999*, 7 = *$60,000 to $69,999*, 8 = *$70,000 to $79,999*, 9 = *$80,000 to $89,999*, 10 = *$90,000 to $99,999*, and 11 = *$100,000 or more*), and marital status (1 = *single,* 2 = *single with partner*, 3 = *married*, 4 = *common*-*law*, 5 = *divorced*, 6 = *separated,* and 7 = *widowed*). Information on child sex and preterm birth status (gestational age of 36 weeks or less at birth) were collected at 4 months postpartum, while number of moves since birth was reported at 3 years postpartum (1 = *haven't moved*, 2 = *moved once*, 3 = *moved twice*, and 4 = *moved three or more times*).

#### 2.2.2. Neighbourhood characteristics

##### 2.2.2.1. Neighbourhood deprivation

The Vancouver Area Neighbourhood Deprivation Index (VANDIX) is a census-based tool that includes both social and economic indicators (39). Participant postal codes from early pregnancy were transformed to geographic coordinates (i.e., latitude and longitude), which were then overlayed on the City of Calgary's neighbourhood boundaries using the spatial join tool in ArcGIS Desktop version 10.6.1 (ESRI, Redlands, CA, USA), and linked with the socioeconomic information from the 2011 National Household Survey census data (40). Following the established method for computing the VANDIX (41), seven indicators (high school completion, university completion, unemployment rate, proportion of lone parent families, average income, home ownership, employment ratio) were weighted (0.250, 0.179, 0.214, 0.143, 0.089, 0.089, 0.036), standardized (z-score), and summed to create a score for each neighbourhood where higher scores represent greater deprivation.

##### 2.2.2.2. Neighbourhood disorder

Neighbourhood disorder was measured objectively using publicly available statistics on disorder (e.g., noise, threats) and crime (e.g., robbery, non-domestic assault) collected from Calgary Police Services' 2010 and 2011 Community Crime Reports, following the Uniform Crime Reporting guidelines (42). The number of crime, physical and social disorder reports were standardized (z-score) and summed to generate a total number of disorder reports per neighbourhood (20, 30).

#### 2.2.3. Lifestyle factors

##### 2.2.3.1. Physical activity

Child physical activity at 3 years of age was assessed by asking participants how much time their child engages in physical activity, such as playing, walking, running, and jumping, on weekdays and weekends. Responses were rated on a six-point scale (where 1 = *none*, 2 = *less than 1 h per day*, 3 = *1 to less than 3 h per day*, 4 = *3 to less than 5 h per day*, 5 = *5 to less than 7 h per day*, and 6 = *7 or more hours per day*). Based on the Canadian Society for Exercise Physiology (CSEP)'s recommendation of at least 180 min of daily physical activity for children aged 3–4 years (43), the variable was dichotomized for analysis, where 0 = less than 3 h of daily physical activity and 1 = 3 h or more of daily physical activity.

##### 2.2.3.2. Parent-Child Reading

At 3 years postpartum, participants were asked “How many minutes each day do you spend sharing books with your child?”. Responses were rated on a four-point scale (where 1 = *0–10 min*, 2 = *11–20 min*, 3 = *21–30 min*, and 6 = *>30 min*). Based on the recommendations for children's vocabulary and school readiness (44), responses were dichotomized for the analysis, where 1 = greater than 20 min per day and 0 = 20 min per day or less.

##### 2.2.3.3. Community resource use

Participants' community resource use at 3 years postpartum was assessed by asking whether they had used or attended (0 = *no*, 1 = *yes*) various community resources or programs in the past year. Listed resources and programs included recreational facilities (e.g., YMCA, leisure centres), libraries, parenting groups, play groups, and childcare centres. Consistent with previous studies using AOF data (45), responses were dichotomized for analysis, where 0 = accessed less than three community resources in the past year and 1 = accessed three or more in the past year.

#### 2.2.4. Child development

Child development at 5 years of age was assessed with the Ages and Stages Questionnaire, Third Edition (46), which is a commonly used, parent-reported and norm-referenced screening tool (47) of developmental progress across five domains: communication, gross motor, fine motor, problem-solving, and personal-social. Subscale scores were summed to determine a total score ranging from 0 to 300 (48), where higher scores were indicative of better developmental outcomes.

### 2.3. Statistical analysis

Descriptive statistics and correlation analyses were conducted using SPSS version 25.0 (IBM, USA). Pearson correlation coefficients were estimated between the neighbourhood characteristics (deprivation, disorder) during pregnancy, lifestyle factors (physical activity, parent-child reading, and community resource use) at 3 years postpartum, and child development (ASQ-3 total score) at age 5. Multilevel modelling, in Mplus version 8.1 (49), was used to test the relationship between neighbourhood characteristics and child development, as well as potential interactions between neighbourhood characteristics and lifestyle factors. As participants in our sample were nested within neighbourhoods, a two-level random model with Bayes estimation (50) was conducted to account for within neighbourhood (level 1) and between-neighbourhood (level 2) effects. Moderation was tested using cross-level interactions by estimating the slope of each lifestyle factor on the ASQ-3 total score at level 1, and then regressing the neighbourhood factors on these slopes at level 2. Any non-significant moderators were removed from the final model. Additionally, several sociodemographic characteristics were considered as control variables including ethnicity, education, household income, preterm status, child sex, and moving (25, 26, 51), and included as level 1 covariates in the final model if they were significantly correlated with the ASQ-3 total score. Missing data were handled using Full Information Maximum Likelihood (FIML), which produces unbiased model parameters (52). Significant effects were determined by a 95% Bayesian credibility interval (BCI) that did not cross zero (53).

## 3. Results

### 3.1. Sample description

After removing those who gave birth to twins (*n *= 36) and those who could not be geocoded (*n *= 907) because they did not provide postal codes or lived outside the city of Calgary boundaries, the final sample consisted of 2,444 participants. Participants were distributed across 192 neighbourhoods, with an average of 12.73 participants each. Overall, 15.17% of the data were missing and covariance coverage ranged from .507–1.00. The sample mostly consisted of pregnant individuals who were married or in common law^1^ relationships (94.9%), identified as European Canadian (77.2%), had attained post-secondary education (76.0%), and an annual household income of greater than $60,000 (82.3%). At 3 years postpartum, most participants had not moved since their child's birth (62.2%). The mean age of participants at <25 weeks gestation was 30.8 years (*SD* = 4.5). Most participants’ children were not born preterm (92.8%) and slightly above half were male (52.4%). Table 1 displays detailed demographic information for the sample.

**Table 1:** Descriptive statistics for study variables (*n *= 2444).

	*n* (*%*)	*M (SD)*	Range
**Demographics (pregnancy)**			
**Ethnicity**			
European Canadian	1,878 (77.2)		
Asian	317 (13.0)		
Latin American	56 (2.3)		
Black	37 (1.5)		
Middle Eastern	36 (1.5)		
Indigenous	21 (0.9)		
Mixed/other	87 (3.6)		
**Marital status**			
Married/common law	2,310 (94.9)		
Single with partner	89 (3.7)		
Single	24 (1.0)		
Divorced/Separated	10 (0.4)		
**Education**			
Completed post-secondary	1,848 (76.0)		
Some post-secondary	338 (13.9)		
Graduated high school	173 (7.1)		
Some elementary or high school	73 (3.0)		
**Annual household income**			
<$10K	27 (1.1)		
$10K-$19K	48 (2.0)		
$20K-$29K	54 (2.3)		
$30K-$39K	77 (3.3)		
$40K-$49K	89 (3.8)		
$50K-$59K	123 (5.2)		
$60K-$69K	128 (5.4)		
$70K-$79K	162 (6.9)		
$80K-$89K	200 (8.5)		
$90K-$99K	197 (8.3)		
≥$100K	1,255 (53.2)		
Child sex (female)	1,164 (47.6)		
Preterm status (≤36 weeks GA at birth)	164 (7.2)		
Moves since birth (≥1 time)^a^	572 (37.8)		
Maternal age (years)		30.78 (4.49)	18–47
**Neighbourhood characteristics (pregnancy)**			
Neighbourhood deprivation		−6.67 (2.86)	−15.64–1.70
Neighbourhood disorder		591.67 (684.44)	1.00–7000.00
**Lifestyle factors (3 years postpartum)**			
Parent-child reading (>20 min daily)	805 (53.1)		
Community resource use (≥ 3 in past year)	993 (65.5)		
Physical activity (≥3 h/day)	953 (62.9)		
**Child development (5 years of age)**			
ASQ-3 total score		274.55 (27.09)	25.00–300.00

*Note*: n, sample size; M, mean; SD, standard deviation; K, thousand; GA, gestational age; ASQ-3, Ages and Stages Questionnaire. Parent-child reading refers to daily minutes participants spent sharing books with their child.

^a^ Measured at 3 years postpartum.

The sociodemographic characteristics of the current sample are consistent with the local Calgary population, where for example the median household income is approximately $97,000, 69.9% have attained post-secondary education, and 78% identify their ethnic origin as North American or European (55). Among participants who could not be geocoded, slightly more identified as European Canadian (80.6%), less were married or in common law^1^ relationships (92.9%), less attained post-secondary education (29.1%), more used ≥3 community resources (75,2%), and were on average older (*M *= 30.78 years).

### 3.2. Descriptive statistics

The mean neighbourhood VANDIX score was −6.67, suggesting that, on average, most participants resided in socioeconomically advantaged neighbourhoods. The number of neighbourhood disorder reports varied considerably across neighbourhoods, ranging from 1 to 7000. At three years postpartum, slightly over half of participants read with their children for greater than 20 min per day (53%), most had accessed 3 or more community resources in the past year (66%), and most children met the CSEP daily physical activity recommendation of 3 h (63%). In terms of development at 5 years of age, children in the sample scored relatively high on the ASQ-3 (*M *= 274.55), given that the maximum possible total score is 300. Few children in the sample scored in either the “monitoring zone” (≤1 *SD* below the mean of the ASQ normative data) or the “referral zone” (≤2 *SD* below the mean of the ASQ normative data) on any of the individual ASQ-3 subscales: communication (7.9%), gross motor (7.7%), fine motor (7.1%), problem solving (2.1%), and personal-social (7.8%). See Table 1 for descriptive statistics for key study variables.

### 3.3. Bivariate correlations

Correlations between neighbourhood variables, lifestyle factors, and child development are reported in Table 2. Neighbourhood deprivation was significantly negatively associated with parent-child reading and community resource use, and positively associated with physical activity. Additionally, neighbourhood disorder was significantly positively associated with physical activity. There was a significant positive relationship between the ASQ-3 total score and parent-child reading. However, the associations between ASQ-3 total score and community resource use and physical activity were not significant.

**Table 2:** Pearson's correlation coefficients.

	1	2	3	4	5	6
***Neighbourhood characteristics***						
1. Deprivation	–					
2. Disorder	.197^b^	–				
***Lifestyle factors***						
3. Parent-child reading	−.111^b^	.008	–			
4. Community resource use	−.111^b^	−.032	.076^b^	–		
5. Physical activity	.085^b^	.052^a^	.090^b^	.010	–	
***Child development***						
6. ASQ-3 total score	−.094^b^	−.048	.077^b^	.018	.038	–

Note. ASQ-3, Ages and Stages Questionnaire.

^a^ Correlation is significant at the 0.05 level (two-tailed).

^b^ Correlation is significant at the 0.01 level (two-tailed).

Correlations between the ASQ-3 total score and potential covariates revealed significant associations with ethnicity (*r *= .078, *p *< .01), education (*r *= .074, *p *< .01), household income (*r *= .091, *p *< .01), child sex (*r *= .175, *p *< .01), and preterm status (*r *= −.127, *p *< .01). These variables were therefore included as covariates in the multilevel analyses. Whether participants had moved or not between birth and 3 years was not associated with ASQ total score (*r* = −.016, *p* = .572) and therefore was not included as a covariate in the multilevel analyses.

### 3.4. Multilevel modelling

The intraclass correlation (ICC) for child development was 0.024, indicating that 2.4% of the variance in total ASQ-3 score was due to variation between neighbourhoods, while 97.6% of the variance was attributable to variation across individuals within neighbourhoods. Parameter estimates from the final multilevel model considering neighbourhood deprivation and disorder during pregnancy as predictors of child development at age 5 are displayed in Table 3. After controlling for level 1 covariates, there was a statistically significant negative association between the VANDIX and ASQ-3 total score, such that higher levels of neighbourhood deprivation during pregnancy were associated with poorer overall child development at age 5. Neighbourhood disorder during pregnancy was not directly associated with total ASQ-3 score.

**Table 3:** Final multilevel model of effects on child development at 5 years of age.

	Estimate, b	95% BCI	*p*-value
Intercept	229.847	166.661, 301.924^b^	<.001
***Covariates (level 1)***			
Child sex	9.726	6.948, 12.533^b^	<.001
Preterm status	−11.404	−17.046, −5.915^b^	<.001
Maternal ethnicity	2.445	−1.242, 6.212	.096
Maternal education	3.513	−.212, 7.269	.032
Household income	3.066	−1.417, 7.659	.091
***Lifestyle factors (level 1)***			
Parent-child reading^a^	–	–	–
Community resource use	−.519	−3.729, 2.625	.375
Physical activity	3.025	−.074, 6.194	.028
***Neighbourhood characteristics (level 2)***			
Deprivation	−.726	−1.344, −.120^b^	.011
Disorder	−.001	−.003,.001	.223
***Moderation effects (level 2)***			
Deprivation on reading slope	−.463	−1.751,.656	.225
Disorder on reading slope	.005	.001,.009^b^	.015

Note: BCI, Bayesian credibility interval.

^a^ Parent-child reading cannot be entered as an independent variable fixed effect since it is turned into a dependent variable in order to define the random effect for the slope on ASQ-3 total score.

^b^ 95% BCI does not cross zero.

In terms of individual- and family- level factors, child sex was found to be significantly associated with child development, with girls having higher ASQ-3 total scores than boys. Preterm birth status was significantly associated with child development, where children who were born preterm had lower ASQ-3 scores than those who were born at <37 weeks gestational age. None of the other individual or family-level variables (ethnicity, education, household income, community resource use, physical activity) were significantly associated with the ASQ-3 total score.

**Figure 1:**
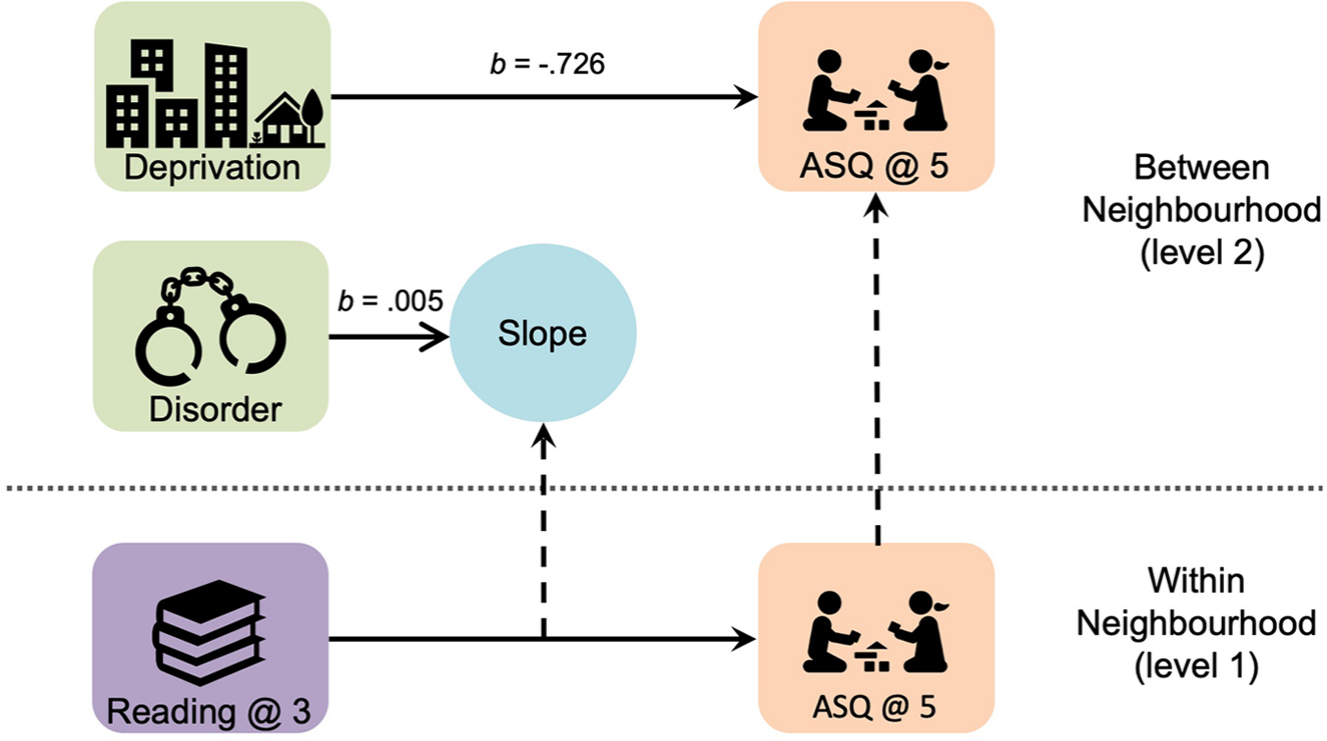
Main and moderation effects of neighbourhood characteristics on child development. Solid arrows represent regression paths, dashed arrows represent parameters brought from the within to the between neighbourhood level. Moderation is represented by the regression of neighbourhood disorder on the slope for Reading to ASQ. ASQ, ages and stages questionnaire.

In terms of moderation effects, there was a statistically significant cross-level interaction where neighbourhood disorder was found to predict a positive slope between parent-child reading and overall child development (see Figure 1). That is, as the amount of neighbourhood disorder increases (i.e., the number of crime and disorder reports), the slope between the ASQ-3 total score and parent-child reading increases. The cross-level interaction of neighborhood deprivation with parent-child reading on development slope was not significant. Additionally, no significant moderation effects were found for community resource use (b_deprivation_**_ _**= .385, 95% CI: −.880, 1.716; b_disorder_**_ _**= .003, 95% CI: −.002,.009) or physical activity (b_deprivation_**_ _**= −.686, 95% CI: −1.970, 0.642; b_disorder_**_ _**= −.003, 95% CI: −.009,.003), therefore these slopes were not included in the final model.

## 4. Discussion

### 4.1. Synthesis of results

The current investigation utilized data from a large Canadian cohort to elucidate the influence of neighbourhood characteristics (deprivation and disorder) during pregnancy on child development, as well as the potential moderation by lifestyle factors (physical activity, parent-child reading, and community resource use). Multilevel analyses indicated that neighbourhood deprivation during pregnancy was associated with poorer child development at age 5 and that parent-child reading may function as a protective factor for child development in the presence of higher neighbourhood disorder. 

The observed association between neighbourhood deprivation and child development, even after controlling for child sex, preterm status, maternal ethnicity, maternal education, and household income, is consistent with extant findings that neighbourhood deprivation is independently associated with developmental delays in preschool- and school-aged children. For example, preschool-aged children living in more deprived neighbourhoods were reported to be 3.15 times more likely to have a speech, language, or communication concern as measured by the ASQ-3 (56). Similarly, after accounting for family-level SES, children in deprived neighbourhoods were found to have concurrently higher levels of behavioural problems and lower cognitive test scores (57). Previous literature has proposed various potential mechanisms for this association, which include reduced access to programs and institutions that promote healthy childhood development, less exposure to highly educated role models in one's neighbourhood, and lower levels of social support and control (58). The current findings are the first to demonstrate associations between prenatal exposure to neighbourhood deprivation and child development, providing further support for the DOHaD hypothesis and pointing to the need for early intervention. Although intervening during pregnancy is proposed to have the largest returns on investments (59), the multilevel nature of social determinants needs to be taken into account. As such, socioeconomic inequalities between neighbourhoods should be addressed as part of policies and programs that promote child and family well-being.

The lack of direct effect of neighbourhood disorder on overall child development at 5 years may reflect a function of age and domain. Neighbourhood disorder has been more frequently linked with development in later childhood and adolescence for conduct problems and mental health outcomes (60–62). Since the ASQ-3 total score captures more motor and cognitive domains and to a lesser extent personal-social outcomes, future research could examine follow-up of specific conduct and mental health outcomes at later ages in the AoF cohort. Interestingly however, there was a moderation effect where as neighbourhood disorder increased during pregnancy, the association between parent-child reading and overall development got stronger. This finding suggests that parents reading with their children more often may buffer the negative impact of prenatal exposure to a neighbourhood with higher disorder. Parent-child reading is proposed to promote child development through various mechanisms such as improved linguistic, interactive, and parental functioning including reduced stress and increased sense of control (63). Parent-child reading interventions are also associated with improved relationship quality (64), which could create a safe space to learn. It is possible that parent-child reading and the related improvement in relationship quality may help to mitigate the impact of prenatal exposure to neighbourhood disorder, such as less safety and more stress (65). Together with previous evidence of the positive psychosocial effects of parent-child reading interventions (64), our results point to this as an important target for public health and community programming (e.g., awareness campaigns, access to books and inclusive reading spaces), particularly for young children living in neighbourhoods with higher levels of disorder.

At the within neighbourhood level two individual characteristics, child sex and preterm birth, were significantly associated with ASQ-3 total scores at five years of age. Consistent with previous research, children reported as male or born before 37 weeks gestation had poorer overall development outcomes. For example, younger gestational age was uniquely associated with increased odds of speech, language, and communication concerns in a statistical model with neighbourhood deprivation (56). Male children have also been observed to have more behavioural problems in a statistical model with neighbourhood social conditions (51). Further research is needed to examine potential moderating effects of individual characteristics to identify who is more vulnerable or resilient to the impact of neighbourhood characteristics on early development (31).

Contrary to expectations, community resource use was not found to have a significant buffering effect against neighbourhood deprivation or disorder, nor was it directly associated with overall child development. These findings may be due to our measure of community resource use mainly capturing participants' own use of community resources rather than their children's direct participation (32, 33). Similarly, no significant moderation effects were observed for physical activity, nor was it significantly associated with overall child development. Although a systematic review indicated that physical activity was associated with improved motor skills and cognitive development in children aged 4–6 years (35), some of the included studies found mixed or no significant effects. Given that parent-report was used to measure children's physical activity and intensity was not distinguished (66), it is possible that we may not have captured the full extent of children's physical activity participation. Future studies in this area could consider using more objective measurements of physical activity, such as standardized questionnaires or accelerometers, as well as capturing variation in the intensity of physical activity that children participate in.

### 4.2. Strengths and limitations

The current investigation utilized a large Canadian cohort study and advanced multilevel analyses to identify early social determinants of individual well-being, and represents a novel examination of prenatal exposure to neighbourhood deprivation on child development. Moreover, potential modifiable protective factors were explored including community resource use, physical activity, and parent-child reading. However, the results of the current investigation should be interpreted within the context of several limitations. Although the use of parent reported measures of child development is common and feasible in population-based birth cohorts (67, 68), potential bias could be mitigated by using multiple informants as well as observational or experimental methods. While the ASQ-3 is a well-validated screening tool for developmental delays, further assessment would be required to examine clinical diagnoses. Given neighborhood deprivation and disorder data were only available for pregnancy, it was not possible to isolate the impact of exposure during this period while accounting for the first five years postpartum. Future research examining cumulative effects of neighbourhood characteristics is warranted. Although several potential confounding variables were included as covariates, it is possible that there are others for which we did not have data such as physical health of children (35). Finally, there was a lack of variability in our sample in terms of sociodemographic and neighbourhood characteristics. In general, the socioeconomic deprivation of neighbourhoods in Calgary is relatively low and residents are more advantaged compared to the larger Canadian population (55). Thus, our findings may not be generalizable to more vulnerable families, or to areas where there are larger differences in socioeconomic deprivation between neighbourhoods.

## 5. Conclusion

Our findings expand previous work on social determinants of well-being by elucidating the association of exposure to neigbourhood deprivation as early as pregnancy, which goes beyond individual and family level factors, with child development. Moreover, the multilevel analysis also identified parent-child reading as a potentially modifiable protective factor in the presence of higher neighbourhood disorder. Neighbourhood-level effects should be considered in the development of policies and community programs that promote family and child well-being.

## Data Availability

The data analyzed in this study is subject to the following licenses/restrictions: The datasets analyzed for this study can be accessed through the University of Calgary subsequent to proposal and ethics review by qualified applicants. Requests to access these datasets should be directed to https://allourfamiliesstudy.com/data-access/.

## Footnotes

^1^Defined as living in a conjugal relationship with a person who is not a married spouse (54).
